# Experimental protocol for evaluation of biomaterials in an *in-vivo* silicone implant coverage

**DOI:** 10.1590/acb396724

**Published:** 2024-09-30

**Authors:** Chenia Frutuoso Silva, Victor de Araújo Felzemburgh, Amanda Dourado Moreno, José Valber Lima Meneses, Aryon de Almeida Barbosa, Isabela Cerqueira Barreto, Fúlvio Borges Miguel

**Affiliations:** 1 Universidade Federal da Bahia – Institute of Health Sciences – Laboratory of Tissue Bioengineering and Biomaterials – Salvador (BA) – Brazil.; 2 Universidade Federal da Bahia – Post-graduation Program in Interactive Processes of the Organs and Systems – Salvador (BA) – Brazil.; 3 Universidade Federal da Bahia – Faculty of Medicine of Bahia – Salvador (BA) – Brazil.; 4 Instituto de Patologia Geral e Cutânea – Salvador (BA) – Brazil.

**Keywords:** Biocompatible Materials, Breast Implants, Models, Theoretical, Rats

## Abstract

**Purpose:**

To describe an experimental surgical model in rats using a dual-plane technique for evaluation of biomaterials in an *in-vivo* silicone implant coverage.

**Methods:**

This study was developed following the ISO 10993-6 standard. In this study, 40 male Wistar rats weighing between 250 and 350 g were used, distributed into two groups: experimental, biomaterial superimposed on the minimammary prosthesis (MP); and control, MP without implantation of the biomaterial, with eight animals at each biological point: 1, 2, 4, 12, and 26 weeks. Thus, at the end of biological points (1, 2, 4, 12, and 26 weeks; n = 8 animals per week), the tissue specimens achieved were fixed in buffered formalin and stained with hematoxylin-eosin.

**Results:**

Macroscopically, throughout the study, no postoperative complications were apparent. In the histological analysis, it was possible to observe the evolution of the inflammatory response, tissue repair, and fibrous capsule during the biological points.

**Conclusions:**

The experimental model described in this study proved to be suitable for evaluating the biomaterial used in the coverage of breast silicone implants.

## Introduction

In recent years, scaffolds have become more prominent in reconstrutive surgeries due to the lack of tissue available for reconstruction. This makes tissue repair highly complex and may culminate in the need to use an autograft, allograft, or xenograft, also known as skin replacement^1^. Biomaterial or therapeutic resources can be used to reduce the risk of postoperative complications when breast implants are used. Postoperative complications that may occur include dehiscence, seroma, hematoma, and necrosis, long hospitalizations with high costs, and contribute to the repair mechanism^2^.

As a result, the implantation of biological scaffolds to provide better repair conditions has led researchers in the field of tissue bioengineering developing biomaterials. These biomaterials aim to replicate the structure and functional features of the extracellular matrix (ECM), to stimulate or replace the biological structure that has been lost. Besides, the biomaterials enable faster tissue repair and better functional and aesthetic results^3^^–^ 5. Among them, those of dermal origin, known as an acellular dermal matrix (ADM), and acellularized bovine pericardium (ABP) stand out.

ADM is a type of allogeneic graft that is made from human cadaver skin. This matrix was initially used in patients with burns and later to fill anatomical defects, correct abdominal walls, perform ophthalmic surgeries, reconstruct the tympanic membrane, and cover silicone implants in the breast^6^. Particularly, it is noteworthy that the application of ECMs has become one of the main options for breast reconstruction with the use of prostheses^7^. Nevertheless, despite having as its main advantage of improving the aesthetic result, it has a high cost and postoperative complications rates.

Therefore, when developing a biomaterial for breast reconstruction surgeries, one should seek to minimize, if possible eliminate, factors that cause postoperative complications and reduce the high cost to make it widely accessible. In this scenario, ABP has been used as an alternative to the use of ADM, because to its wide availability and accessibility, biological properties, and physicochemical characteristics that favor tissue repair more quickly and with less risk of postoperative complications^8^^–^ 12.

Nonetheless, a fundamental condition for using biomaterials is to determine their safety and efficacy in *in-vivo* experimental tests, evaluating and understanding the observed biological responses as a function of interaction with host tissue, before employing them in clinical therapies or tissue replacements^13^. In these studies, animal models are used to mimic the pathogenesis of some diseases to understand the physiopathology; develop vaccines and diagnostic tests, test drugs and medications; evaluate new surgical and regenerative techniques, medical devices, and biomaterials, to conduct translational research and future clinical applications. The primary importance of experimental protocols in this context lies in their support for scientific and technological advances in various areas of knowledge^13^^–^ 15.

Concerning these studies, the International Organization for Standardization (ISO) has defined standards to launch international standards that establish *in-vivo* protocols for the biological evaluation of medical devices, based on the analysis of the local response after implementation (ISO 10993-6)^16^. According to biomaterial evaluation protocols, subcutaneous tissue is the most suitable for testing new materials in terms of biocompatibility, immunogenicity, biological behavior, and integration^13^^,^ 17 ^,^ 18. According to ISO 10993-6^16^, the region located beneath the *panniculus carnosus* muscle is particularly suitable for evaluating polymeric materials.

In the evaluation process of non-degradable and non-resorbable materials, ISO 10993-6^16^ recommends analyzing short-term responses for a period of one to four weeks. Concerning long-term responses, tests must be carried out for 12 weeks or more, as the reaction resulting from the surgical procedure can be difficult to distinguish from the tissue response caused by the implantation of the biomaterial.

Based on the aforementioned information, this study aimed to develop an experimental protocol to describe an experimental surgical model in rats using a dual-plane technique for the evaluation of biomaterials in an *in-vivo* silicone implant coverage.

## Methods

### Ethical considerations

This study was conducted after approval by the Ethics Committee on the Use of Animals of the Health Sciences Institute of the Universidade Federal of Bahia (Protocols no. 115/2017 and 4715160421/2021), following the current regulations on animal experimentation. Besides, this study has been conducted according to ISO 10993-6^16^ standard.

### Experimental surgical protocol

In this study, 40 male Wistar rats weighing between 250 and 350 g were used, distributed into two groups:

- Experimental group (EG): biomaterial (ABP) superimposed on the minimammary prosthesis (MP);
- Control group (CG): MP without the implantation of the biomaterial.

There were eight animals at each biological point: 1, 2, 4, 12, and 26 weeks. To perform the surgical procedure, the researchers administered an intraperitoneal anesthesia to the rats, following the method described by Damy et al.^19^, using a combination of ketamine hydrochloride (75 mg/kg) and xylazine hydrochloride (5 mg/kg). Subsequently, trichotomy and antisepsis of the dorsum region of the animals were performed with 2% alcoholic chlorhexidine.

In compliance with the guidelines of the 3Rs program (Replacement, Reduction, and Refinement), a MP was implanted in the submuscular plane of all animals, on both sides of the back: left (EG) and right (CG). For this purpose, two areas of the skin (in the EG and CG) were delimited from the mid-sagittal line and a horizontal line at the height of the lower costal ridge, as described by Schmitz et al.^20^ and Kafejian et al.^21^.

**Figure 1:**
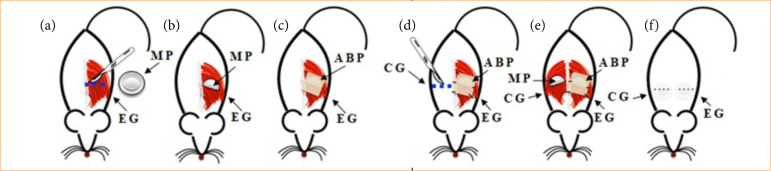
Schematic representation of the implantation of minimammary prothesis (MP) and acellularized bovine pericardium (ABP) on the animal’s back in frontal view. **(a)** Delimitation and horizontal incision in the skin on the experimental group (EG) side; **(b)** inclusion of the MP in the submuscular plane on the EG side; **(c)** ABP matrix overlapped on the MP-muscle assembly; **(d)** ABP suture on the EG side, delimitation, and incision of the skin on the CG side; **(e)** inclusion of MP on the control group (CG) side, without ABP coverage; **(f)** suturing of the skin flap on both sides of the animal’s back.

Source: Elaborated by the authors.

**Figure 2:**
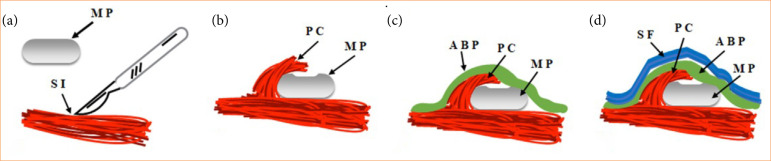
Schematic lateral view of minimammary prothesis (MP) and acellularized bovine pericardium (ABP) after submuscular implantation in the animal. **(a)** MP and surgical incision (SI) in the muscular plane; **(b)** MP partially overlapped by the *panniculus carnosus* (PC); **(c)** overlapping of the MP-PC set with the ABP matrix; **(d)** skin flap (SF) repositioned after the implementation of ABP.

Source: Elaborated by the authors.

Subsequently, a horizontal incision in the skin was made on each side of the back, approximately 1-cm long (Fig. 1a). Then, the subcutaneous tissue was incised and divulsed on both sides, followed by an incision in the muscular plane of the *panniculus carnosus* (Fig. 2a) to include the textured silicone MP with a round shape (2 mL) (Silimed) in the submuscular region (Fig. 1b). Once this was accomplished, the muscle layer was coaptated to partially cover the MP (Fig. 2b).

On the EG, the MP was overlapped with an ABP matrix, which covered the entire MP-muscle set (Figs. 1c and 2c), and the fixation was performed with four interrupted stitches with 5 nylon thread (Fig. 1d). On the CG, the same procedures were performed, except for the implantation of the ABP matrix on the MP (Fig. 1e). At the end, the skin flaps (SF) on both sides of the animal’s dorsum were repositioned (Fig. 2d) and sutured with interrupted stitches with 5 nylon thread (Fig. 1f).

### Steps of the surgical procedure

**Figure 3:**
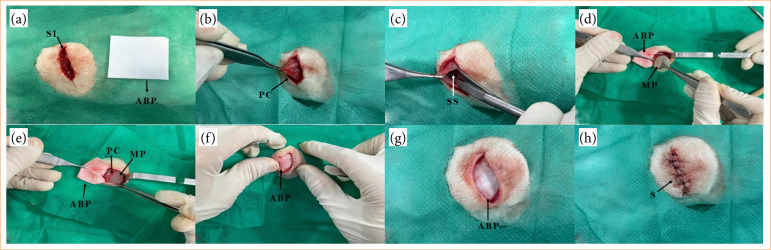
Stages of the surgical procedure for implantation of the minimammary prothesis (MP) and acellularized bovine pericardium (ABP) matrix. **(a)** surgical incision (SI); **(b)** *panniculus carnosus* (PC) muscle; **(c)** preparation of the submuscular site (SS); **(d)** implementation of MP and ABP in the SS; **(e)** MP partially covered by the PC; **(f)** ABP matrix attached to the underlying muscle tissue; **(g)** overlapping of the MP-PC set by the ABP matrix; **(h)** suture **(S)** of the skin flap with interrupted stitches.

Source: Elaborated by the authors.

The surgical procedure for the implantation of the MP and the ABP matrix can be seen in Fig. 3. Initially, a cutaneous incision was made on each side of the animal’s dorsum (Fig. 3a), followed by divulsion of the adjacent subcutaneous tissue to access the *panniculus carnosus* (Fig. 3b) and to make the submuscular site (Fig. 3c) for implantation of the MP and the ABP matrix (Fig. 3d) so that the *panniculus carnosus* was partially overlapped with the MP (Fig. 3e). Subsequently, the four ends of the ABP matrix were sutured to the surrounding muscle tissue (Fig. 3f) to fix the biomaterial, ABP, overlapped on the MP-muscle set, throughout the study (Fig. 3g). Finally, the SF was repositioned and sutured with interrupted stitches using 5 nylon thread (Fig. 3h).

### Obtaining tissue samples and laboratory stage

**Figure 4:**
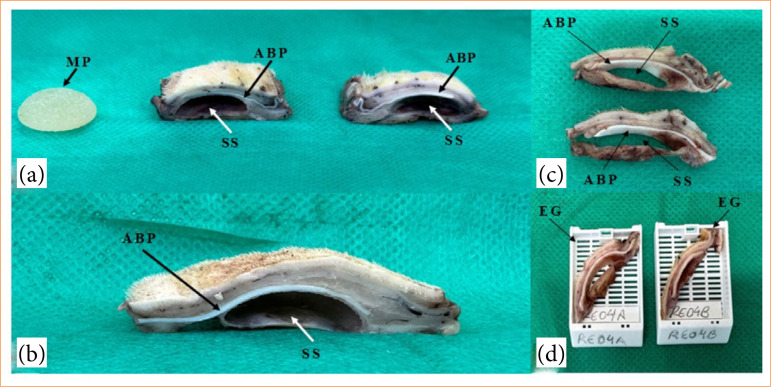
Macroscopic view of experimental group (EG) specimens after fixation. **(a)** Silicone minimammary prothesis (MP) and EG specimens after sectioning in half. **(b)** Submuscular site and acellularized bovine pericardium (ABP). **(c)** Lateral view of the tissue fragments of the EG. **(d)** Tissue fragments of the EG included in the cassette. Note the submuscular site (SS), previously occupied by the MP and covered by the ABP matrix.

Source: Elaborated by the authors.

Following the recommendations of ISO 10993-6^16^, after the biological points of 1, 2, 4, 12, and 26 weeks, the animals were euthanized with an intraperitoneal lethal injection of ketamine hydrochloride (300 mg/kg) and xylazine hydrochloride (30 mg/kg). Afterward, tissue specimens were achieved (Fig. 4), with a margin of 1 cm from the edge of the MP and depth below the muscle plane, to include the PC in the tissue sample. Then, the specimens were fixed in buffered 4% formaldehyde for 48 hours. After this period, MP was removed from all tissue samples (EG and CG), and the specimens were sectioned in half (Figs. 4a and 4b).

The fragments achieved from each specimen (Fig. 4c) were sent for routine histological processing (Fig. 4d), embedded in paraffin, cut into 5-μm thick sections and stained with hematoxylin and eosin (HE). The histological sections were examined by light microscopy (DM6B – Leica Biosystems Nussloch GmbH, Germany) and photographed with a DFC 7000T camera (Leica) and LAS V.4.12 Leica Application Suit (Leica) software.

## Results

None of the animals died or exhibited postoperative complications during the study period. In all biological points, in the two groups studied, macroscopically, the animals did not present local postoperative complications, such as hematoma, infection, abscess, seroma, wound dehiscence, extrusion of the MP and ABP, and capsular contracture (CC). During the experiment, the animals kept their regular behavior and increased their weight during the biological points.

**Figure 5:**
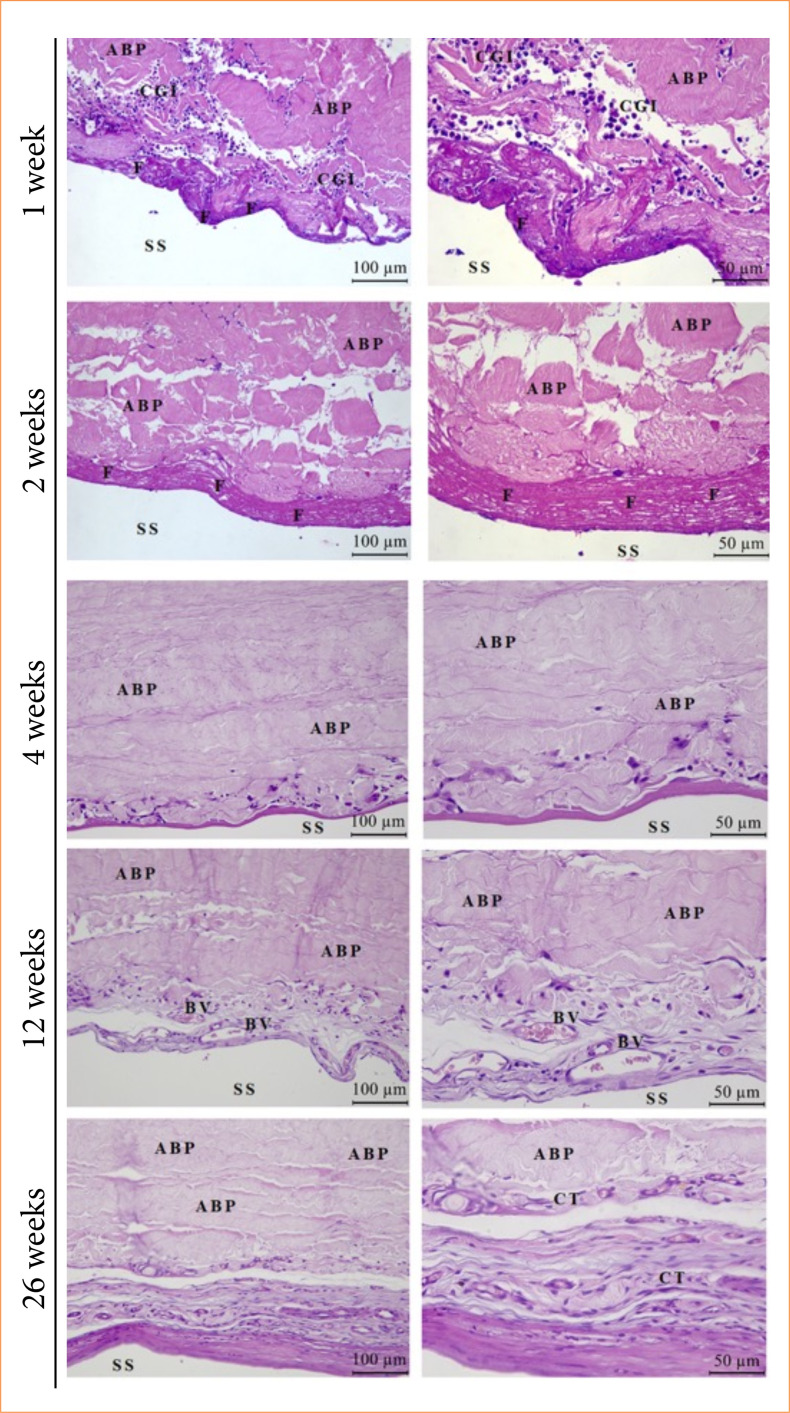
Photomicrographs of the experimental group at the biological points at 1, 2, 4, 12 and 26 weeks (n = 8 animals per week). The tissue reaction is observed through the chronic granulomatous inflammatory response and fibrous capsule formation.

CGI: chronic granulomatous inflammation; F: fibrin; ABP: acellularized bovine pericardium; SS: submuscular site; CT: connective tissue; BV: blood vessel. Source: Elaborated by the authors.

**Figure 6:**
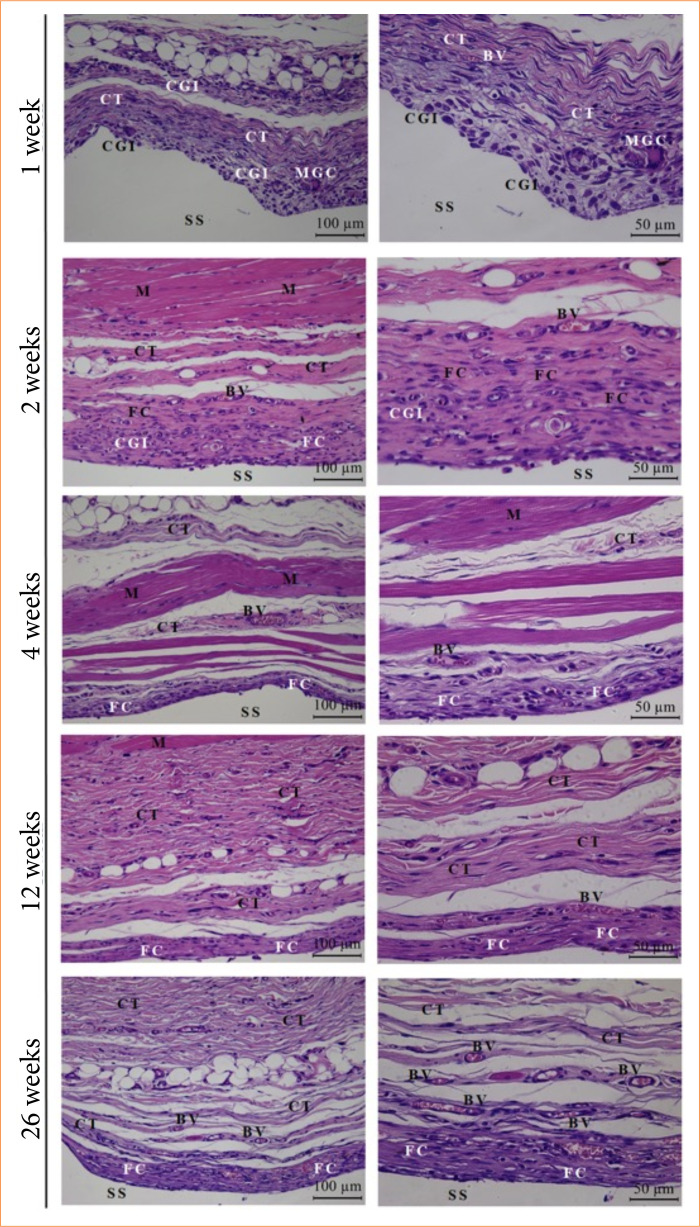
Photomicrographs of the control group at the biological points at 1, 2, 4, 12 and 26 weeks (n = 8 animals per week). Tissue response is observed through CGI and FC formation.

FC: fibrous capsule; CGI: chronic granulomatous inflammation; M: muscle; SS: submuscular site; CT: connective tissue; BV: blood vessel; MGC: multinucleated giant cells. Source: Elaborated by the authors.

In the histopathological analysis, it was possible to verify, in the biological points considered, the evolution of the tissue reaction by observing the inflammatory response, the repair, and the fibrous capsule formed after the implantation of MP and ABP. In the first three analysis periods, there was a moderate chronic granulomatous inflammatory response in the EG and CG, including edema, granulation tissue, and fibrous capsule formation. Particularly at 12 weeks, the group that received ABP had a greater pronounced expression of these last two findings. At the final biological point, there were few inflammatory cells, the organization of the fibrous capsule in the two groups studied, and the biointegration of ABP with host tissue in the EG (Figs. 5 and 6).

## Discussion

Advances in the development of biomaterials require experimental research in to evaluate these materials in terms of biological response, with a view to future clinical applications. In this context, *in-vivo* assays are the most appropriate because they show the tissue reaction and the inflammatory response, which support the evaluation of the biocompatibility and biodegradation of these materials. To this end, such studies need to comply with the ethical and animal welfare guidelines and recommendations delineated by the regulatory agencies in each country, and consider the observation periods that justify the purpose of the experimental research.

As a result, laws, controls, and inspections have become increasingly robust with the development of more specific regulations for each purpose of investigation and type of research. In this way, the protocols and results achieved are more accurate and avoid the need to perform new experiments with the same objectives, enable reproducibility when necessary, and present valid data for later extrapolation in translational research^13^^,^ 15.

Therefore, this study aimed to describe in detail an experimental surgical protocol using a dual-plane technique for the evaluation of biomaterials in the breast silicone implant coverage in a preclinical model in rats, following the recommendations of ISO 10993-6^16^. The purpose of the description and modification to a dual-plane technique was to provide an interface between the ABP, MP, and muscle tissue, similar to what is performed in the surgical technique with human beings. This is the essential aspect in the innovation of the experimental surgical protocol presented, since all other biomaterial implantation protocols, in animal models, are in the subcutaneous plane. It was chosen to use this animal model, since it is easy to breed and maintain, available and affordable, and it has faster tissue repair than other species used in experiments^22^ and shares physiological similarities with humans^23^.

The surgical protocol described in this study proved to be adequate, since it made it possible to analyze the evolution of the inflammatory response, tissue repair, and fibrous capsule. The chronic granulomatous inflammation observed in both groups was shown to be of moderate intensity at the beginning, and at the end was scarce with sparse mononuclear cells in both the EG and the CG, similar to the findings of several studies that also evaluated different biomaterials with different purposes^24^^–^ 31. However, it is known that, immediately after implantation, the inflammatory response is acute due to tissue lesions caused by the surgical procedure^32^ and activation of inflammatory cells with consequent secretion of pro-inflammatory cytokines^33^. When there is biocompatibility, the permanence of the biomaterial, permeating the host tissues in the region of the surgical site, stimulates the evolution of this response to chronic inflammation^24^^,^ 32 ^,^ 34 ^–^ 37 and repair; or function performance for which the material was designed.

The development of an exacerbated inflammatory response may culminate in the expulsion of the biomaterial or excessive deposition of fibrous tissue, thicker fibrous capsule formation and, consequently, cause CC, which is considered a postoperative complication that functionally and aesthetically compromises the outcome^29^, in cases in which silicone implantation occurs. Thus, to reduce or even avoid this phenomenon, different biomaterials have been used in recent years as an interface between the silicone implant and the surgical site, in an attempt to prevent direct contact with the host’s tissues. The EG showed thinning of the fibrous capsule at the final biological point, in line with the findings of Schmitz et al.^20^, Bernardini et al.^29^, and Ludolph et al.^38^, who also evaluated biomaterials in the silicone coverage.

Such results will be of great importance for breast reconstruction surgeries with silicone implant coverage, as they suggest a potential advantage in decreasing possible postoperative complications, particularly in decreasing fibrous capsule formation and CC. The results of this study may be also beneficial for translational research.

## Conclusion

The surgical protocol described in this study mimicked breast reconstruction with silicone implants associated with ABP collagen matrix coverage, except for the site of MP implantation. The results achieved made it possible to understand the tissue reaction and the evolution of the inflammatory response and repair of the fibrous capsule over the period of 26 weeks, in addition to biointegration with host tissue. These results will support the development of experimental studies in the field of tissue bioengineering.

## Data Availability

All data sets were generated or analyzed in the current study.
